# West Texas Medical Association

**Published:** 1902-08

**Authors:** W. B. Russ

**Affiliations:** Secretary


### West Texas Medical Association.

The West Texas Medical Association held a special mid-summer meeting at New Braunfels on the afternoon and evening of July 31st. This meeting was called at the invitation of Drs. Garwood, Leonards and Noster, of that town.

About forty physicians were present, including visitors, the following being among the places represented: San Antonio, Austin, San Marcos, Beeville, Cuero, Corpus Christi, Hunter, Seguin, Marion, Devine, Yoakum and Galveston.

The afternoon session was called to order at 3 o'clock, and papers were read by Drs. G. Graham Watts, M. L. Graves, B. F. Kingsley, G. H. Moody and others. Besides the usual scientific features the program included a drive through Landa's Park, followed by a luncheon in the park pavilion, and later by a boat ride on the Comal river on board the steamer "Wilhelm der Grosse."

Plans are being made to hold a similar meeting at Corpus Christi during the last week in September.

W. B. RUSS,
Secretary.

